# Reply to Interrogator on the Best Mode of Extracting the Placenta

**Published:** 1816-11

**Authors:** T. Purton

**Affiliations:** Member of the Royal College of Surgeons, &c. &c.


*For the London Medical and Physical Journal.*

*Reply to Interrogator on the best Mode of extracting the Placenta;* by T. Purton, Member of the Royal College of Surgeons, &c. &c.

IN the course of nearly thirty years of very considerable midwifery practice, I have only met with one case of *central* presentation of the placenta; in that instance there could be no hesitation on the part of the practitioner of the best mode of procedure. The whole surface of the placenta being attached firmly, excepting that part immediately opposed to the os uteris, passing the hand through that part already detached would certainly be attended with less danger, particularly to the mother, than attempting to separate more.

In another case, where a considerable portion of the placenta extended over the os uteri, I readily passed my hand without tearing the cake.

There can be no doubt in my mind of the propriety, as well as greater safety both to the mother and child, to rupture the placenta in preference to déstroying it; I mean in those cases where the attachment of the placenta over the os uteri is situated so nearly its centre, that we consequently must risk the mother's life, more by attempting any farther separation, than by pérforating its substance.

*Alcester; October* 7, 1816.

