# Erratum

**Published:** 1817-02

**Authors:** 


Erratum.—In page 364, line 11, of the last volume, for *destroying* read *detaching.*

